# Study on the Characteristics of MBN and MAE Signals in P92 Steel

**DOI:** 10.3390/ma19112311

**Published:** 2026-05-29

**Authors:** Ziyi Huang, Xiaochu Pang, Xinnan Zheng, Saibo She, Xufei Liu, Wuliang Yin, Lisha Peng

**Affiliations:** 1 Department of Electrical and Electronic Engineering, The University of Manchester, Manchester M13 9PL, UK; ziyi.huang-7@postgrad.manchester.ac.uk (Z.H.); xiaochu.pang@postgrad.manchester.ac.uk (X.P.); xinnan.zheng@ieee.org (X.Z.); saibo.she@postgrad.manchester.ac.uk (S.S.); 2 Department of Electrical Engineering, Tsinghua University, Beijing 100084, China; liuxf22@mails.tsinghua.edu.cn

**Keywords:** creep damage evaluation, magneto-acoustic emission, magnetic Barkhausen noise

## Abstract

The demand for efficient combustion in boilers drives the development of ultra-supercritical power plants. P92 steel pressure, and pipelines operate in high-temperature and high-pressure environments and are prone to high-temperature creep damage. Non-destructive testing is a key method to ensure the safety of the pipe. However, existing non-destructive testing methods are difficult to achieve non-destructive detection of creep damage. Creep damage affects magnetic Barkhausen noise (MBN) and magneto-acoustic emission (MAE) signals; therefore, it is possible to evaluate creep damage using these signals. This article first establishes a theoretical model for MBN and MAE. Afterward, the influence of magnetizing waveform, amplitude, and frequency on MBN and MAE signals was studied through experiments. Finally, by analyzing the characteristics of MBN and MAE signals, the optimal magnetization conditions and signal characteristic parameters for detecting creep damage using MBN and MAE signals were determined. The experimental results also confirmed the correctness of the theoretical model.

## 1. Introduction

With the continuous growth of global energy demand and the promotion of low-carbon development goals, Ultra Supercritical (USC) thermal power plants have become an important development direction for modern thermal power generation due to their high efficiency and low emissions characteristics [1]. In such plants, key high-temperature components usually serve for a long time in high-temperature and high-stress environments above 600 °C and 25 MPa, and the stability of their material properties directly affects the safety and economy of plant operation [2]. Therefore, in-depth research on the service behavior of high-temperature structural materials is of great engineering significance [3].

P92 steel, as a typical 9% Cr ferrite martensitic heat-resistant steel, is widely used in key parts such as main steam pipelines and manifolds due to its excellent creep resistance and structural stability. Previous studies have shown that the internal structure of P92 steel undergoes significant evolution during long-term high-temperature service. For example, the martensitic Flat noodles gradually recover and coarsen, leading to the weakening of the strengthening effect [4]. Meanwhile, the decrease in dislocation density will significantly weaken the high-temperature strength of the material [5]. In addition, the coarsening of M carbides and Laves phases is considered an important factor leading to the degradation of creep performance [6]. Recent studies have further indicated that there are differences in the evolution paths of tissues under different stress conditions, which affect the creep behavior of materials. Phase transformation kinetics, grain growth, and carbide/precipitate evolution under different stress states have been reported to significantly alter creep resistance in Cr–Mo–V steels [7].

During creep damage, the material undergoes complex microstructural evolution internally. The relevant constitutive models indicate a significant coupling relationship between creep deformation and microscopic damage evolution [8]. Under high temperature stress, grain boundary slip gradually increases and promotes the nucleation and growth of voids [9]. As the service time prolongs, grain boundary voids continue to accumulate and connect, ultimately leading to material fracture [10]. In addition, welded joints often become the preferred area for creep damage due to uneven microstructure and residual stress concentration [11]. Recent studies have also shown that welding areas exhibit more complex damage mechanisms and higher failure risks after long-term service.

The traditional creep damage assessment methods mainly rely on metallographic analysis and mechanical property testing, which usually require sampling and have certain destructive properties, making it difficult to achieve online monitoring [12]. Although acoustic emission technology can achieve real-time monitoring to some extent, its ability to respond to early microstructural changes is still limited [13]. Therefore, developing highly sensitive and non-destructive detection technologies is of great significance for achieving early damage identification.

In recent years, electromagnetic non-destructive testing technology has received widespread attention due to its lack of coupling media and high sensitivity to microstructural changes in ferromagnetic materials. The relevant review systematically summarizes the potential application of magnetic detection methods in material performance evaluation [14]. On this basis, Magnetic Barkhausen Noise (MBN) technology has been widely used to characterize microdefects inside materials, and its signal characteristics can effectively reflect dislocation density and precipitation phase changes [15]. Further research has shown that changes in stress state also significantly affect the MBN signal response characteristics [16]. In recent years, with the development of signal processing and machine learning methods, the application of MBN technology in stress assessment and damage identification has been further expanded [17].

From a mechanistic perspective, the MBN signal originates from the discontinuous transitions of magnetic domain walls under the influence of an external magnetic field, and its behavior is influenced by microscopic obstacles such as dislocations, precipitates, and grain boundaries. Research has shown that the coupling effect of stress and microstructural changes can significantly alter the behavior of magnetic domain motion, thereby affecting signal characteristics [18]. On this basis, MBN technology has gradually been applied to high-temperature creep damage assessment and has made certain progress [19].

On the other hand, magnetoacoustic emission technology is based on the magnetostriction effect, which causes material micro-deformation and releases elastic wave signals through magnetic domain motion during magnetization. Compared to MBN, magneto-acoustic emission (MAE) is more sensitive to microcrack initiation and stress concentration areas [20]. The latest review studies indicate that MAE technology has good application prospects in the field of non-destructive testing, especially for the identification of damage in materials in the later stage [21].

It should be pointed out that both MBN and MAE signals are highly sensitive to magnetizing parameters [22]. Previous studies have shown that parameters such as magnetizing waveform, amplitude, and frequency significantly affect the magnetic domain motion behavior and signal characteristic distribution [23]. However, the current research on the response laws of two types of signals under multi-parameter coupling for P92 steel material is still relatively limited.

Therefore, this article takes P92 steel as the research object and systematically studies the influence of different magnetizing waveforms, amplitudes, and frequencies on the characteristics of MBN and MAE signals. Through multi-parameter experimental analysis, the intrinsic relationship between magnetizing conditions and electromagnetic response is revealed, and the combination of detection parameters is optimized to improve the sensitivity and reliability of creep damage assessment. This study can provide a theoretical basis and technical support for the engineering application of electromagnetic non-destructive testing technology in predicting the service life of high-temperature components.

## 2. Model of the MBN and MAE

### 2.1. Basic Magnetization Model

P92 steel is a polycrystalline ferromagnetic material. During the magnetization process, the energy of the magnetic domains inside P92 will change, but at any time, the internal magnetic domains will always be in a state of energy minimization. Magnetic domain energy includes magnetic crystal anisotropy energy, magnetoelastic energy, domain wall energy, and magnetic intrinsic energy, etc. Among the energy components considered, creep damage exerts the strongest influence on magnetic domain wall energy and also affects magnetoelastic energy through changes in local stress concentration and microstructural evolution [24]. In addition, the original contribution of this work lies in the experimental design, signal acquisition strategy, and statistical evaluation of the MBN and MAE responses under the selected testing conditions. Unlike previous studies that mainly reported qualitative trends, this work provides a systematic comparison of the measured signal characteristics and establishes a reproducible analysis workflow for the investigated material.

A hysteresis model based on domain-wall motion is suitable for describing the phenomenon of MBN and MAE in multi-domain specimens. The effect of domain rotation in the model is considered by establishing the relationship between energy dissipation and change in magnetization [25].

The change in energy through wall pinning of a 180° domain wall under a magnetic field *H* can be calculated by Equation (1).

(1) $$dE=-2\mu_{0}M_{S}HAdx$$

where d*E* is energy loss, $\mu_{0}$ is the permeability of free space, *M_s_* is saturation magnetization, *A* is domain wall cross-sectional area and d*x* is moving distance. The pinning energy $\varepsilon_{pin}$ is proportional to the change in energy per unit volume caused by moving the domain wall.

(2) $$\varepsilon_{pin}=\frac{1}{2}\varepsilon_{\pi }(1-\cos\phi )$$

where ϕ is the angle between the moments in the neighboring domains, and $\mu_{0}\varepsilon_{\pi }$ is the pinning energy for a 180° wall.

If there are n pinning sites per unit volume, the energy dissipated by moving a 180° domain wall through a volume depends on the pinning energy. The energy dissipated is

(3) $$dE_{loss}=\mu_{0}n\varepsilon_{\pi }Adx$$

where *n* is the number of pinning sites per unit volume, *A*d*x* is the volume swept out by the wall as it moves.

The change in the magnetic moment will be

(4) $$dm=2M_{s}Adx$$

Therefore, the energy loss per cubic meter will be

(5) $$dE_{loss}=\frac{\mu_{0}n\varepsilon_{\pi }dM}{2M_{s}V}=\frac{\mu_{0}N\varepsilon_{\pi }dM}{2M_{s}}$$

where *V* is the volume, replacing *k* with the loss coefficient, the energy loss can be simplified as

(6) $$dE_{loss}=\mu_{0}kdM$$

which shows that the energy loss per unit volume in magnetizing is proportional to the change in magnetization.

The Barkhausen activity is proportional to the product of the differential susceptibility $\frac{dM_{irr}}{dt}$ and the rate of change in the field $\frac{dH}{dt}$.

(7) $$\frac{dM}{dt}=\gamma \frac{dM_{irr}}{dH}\frac{dH}{dt}$$

where γ is the fraction of the irreversible magnetization change, *M_irr_* is the irreversible magnetization. The term γ can be determined by

(8) $$\gamma =\frac{d}{dM_{irr}}(NM_{\mathrm{disc}})$$

where *N* is the number of Barkhausen events, and $M_{disc}$ is the average size of the Barkhausen events. This method of analyzing the Barkhausen signals in a stochastic model is based on the fact that the event size $M_{disc}$ and the number of events *N* vary in a random manner.

The expression for Barkhausen activity becomes

(9) $$\frac{dM}{dt}=\frac{dM_{irr}}{dH}\frac{dH}{dt}(N\frac{dM_{disc}}{dM_{irr}}+M_{disc}\frac{dN}{dM_{irr}})$$

The size of the Barkhausen jumps $M_{disc}$ is weakly related to the irreversible change in magnetization. The number of events *N* is approximately linear to *M_irr_*. Therefore, the magnetization rate is approximated as

(10) $$\frac{dM}{dt}=\frac{dM_{irr}}{dH}\frac{dH}{dt}M_{disc}\frac{dN}{dM_{irr}}$$

Most Barkhausen activity occurs close to the zero of the magnetic field *H*, where the slope $\frac{dM}{dH}$ of the magnetization curve reaches a maximum. Therefore, for a fixed rate of change in magnetic field $\frac{dH}{dt}$, the rate of magnetization change $\frac{dM}{dt}$ reaches a maximum at the zero of magnetic field *H*. Of course, the creep defect changes the values and the positions of the peaks of the MBN and MAE signals.

### 2.2. Magnetostriation Mechanism in MAE

Magnetostriction refers to the phenomenon where ferromagnetic materials undergo macroscopic dimensional/shape changes upon magnetization in a magnetic field under zero external stress, with its core mechanism lying in the coupling between the magnetization state and lattice strain. For polycrystalline ferrite-martensite heat-resistant steels like P92 steel, the microscopic contributions to magnetostriction primarily arise from the pinning of domain walls by dislocations and precipitates, as well as the domain rotation constraints imposed by grain boundaries. Combining the magneto-mechanical effect model [22], magnetostriction can be correlated with the magnetization curve through equivalent stress-magnetic fields. Under low magnetic fields, magnetostriction exhibits a quadratic relationship with magnetization intensity:

(11) $$\lambda =bM^{2}$$

where *b* is the material constant (related to the Cr, W, and V content in P92 steel), and *M* represents the magnetization intensity. The magnetostriction strain introduces an equivalent magnetic field that is superimposed on the external magnetic field to influence magnetization. The equivalent magnetic field *H_σ_* under the influence of stress *σ* is

(12) $$H_{\sigma }=\frac{3\sigma }{2\mu_{0}}\frac{d\lambda }{dM}=\frac{3b\sigma }{\mu_{0}}M$$

The greater the stress *σ*, the stronger the magnetostriction modulation on the magnetization. When there is no internal field coupling (*αM* = 0), the correction to the hysteresis-free magnetization curve, *M_an_*, due to magnetostriction, satisfies the Frohlich-Kennelly relation.

(13) $$M_{an}\left(H\right)=\frac{\beta \left(H+\frac{3\sigma }{2\mu_{0}}\frac{d\lambda }{dM}\right)}{1+\beta \left(H+\frac{3\sigma }{2\mu_{0}}\frac{d\lambda }{dM}\right)}M_{s}$$

where *β* is the hysteresis-free magnetization coefficient, and *M_s_* is the saturation magnetization.

In summary, when an alternating magnetic field drives domain wall motion, the instantaneous micro-deformation (d*λ*/d*t*) generates elastic waves (acoustic waves), which are captured by the sensor as MAE signals. The MAE amplitude is proportional to the magnetostriction strain rate (d*λ*/d*t*), while the MAE pulse count correlates with the number of discrete micro-deformation events. The linear magnetic field variation in triangular waves continuously accumulates strain, resulting in a pulse count slightly higher than that of sine waves. The MAE bandwidth is affected by the frequency decay of elastic waves. Although square waves contain rich higher-order harmonics, their high-frequency components decay rapidly, leading to a narrower bandwidth than sine waves. Therefore, MAE represents the macroscopic manifestation of magnetostriction.

## 3. Experimental System

### 3.1. Specimen

This experiment selected P92 steel as the research object and designed high-temperature creep standard specimens in accordance with the Chinese national standard [23]. The sample adopts a dumbbell-shaped structure, with an effective working section length of 100 ± 0.2 mm, a diameter of 10 mm, a transition arc radius of 25 mm, and a total length of 226 mm. This design ensures that stress is concentrated in the effective working section of the sample during high-temperature creep, avoiding premature failure in the transition zone and ensuring the reliability of experimental data.

P92 steel, as a typical chromium molybdenum vanadium heat-resistant steel, strictly follows industrial application standards in its chemical composition. The main element composition and mass fraction are shown in Table 1.

This component design ensures the durability and creep resistance of the material at high temperatures of 550–620 °C through the synergistic effect of solid solution strengthening of tungsten and molybdenum elements and precipitation strengthening of vanadium and niobium elements.

**Figure 1:**
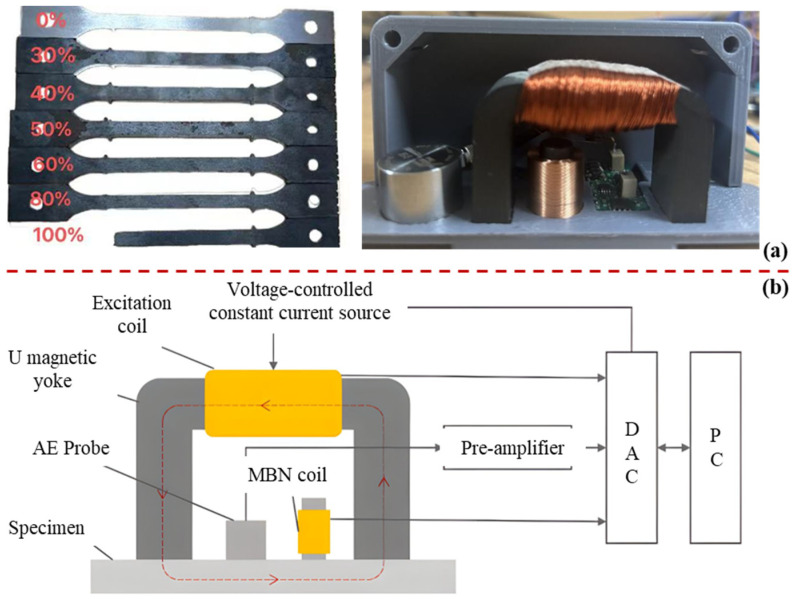
Magneto-acoustic composite detection: (**a**) Original specimen, creep damage specimen; (**b**) overall framework of the magneto-acoustic composite detection device.

To investigate the effects of different degrees of creep damage on MAE and MBN signals, a series of creep tests was conducted at 650 °C and 100 MPa. By controlling the creep time, samples with different proportions of life were prepared, including original undamaged samples (0% life) and 30%, 40%, 50%, 60%, 80% and 100% life interrupted samples (Figure 1), which mainly consists of an excitation module, a joint signal pickup module, a signal conditioning and acquisition module, and a host computer software. The signal generator or signal acquisition card generates excitation signals such as sine waves, triangular waves, square waves, etc., which are power amplified to drive the excitation coil to produce an alternating excitation magnetic field. When the sample is plate-shaped or has a large size, a U-shaped yoke is usually used for excitation. A 1 Ω resistor is connected in series with the excitation coil to monitor the amplitude of the excitation current. Magnetic sensors, acoustic emission probes, magnetic Barkhausen noise induction coils, etc., are placed on the surface of the tested piece to acquire magnetic-acoustic signals such as tangential magnetic field, MAE, MBN, etc. The acquired signals are filtered and processed further in the host computer software.

The interrupted sample retained the microstructure state of the corresponding creep stage through rapid cooling, and local microcracks with a length of about 200 μm were observed at the fracture surface of the fractured sample, which is consistent with the typical failure characteristics of high-temperature creep of P92 steel. In the detection system, the data acquisition card is an ART acquisition card USB2872, the voltage-controlled constant current source is an OPA549 voltage-controlled constant current source module, and the acoustic emission probe is a Qingcheng G150 acoustic emission sensor (QAWRUMS Ltd., Guangzhou, China), which is connected to a PAS preamplifier. The switching power supply and positive and negative voltage modules provide a DC regulated power supply for the acquisition card and various modules.

### 3.2. Experimental System Construction

**Figure 2:**
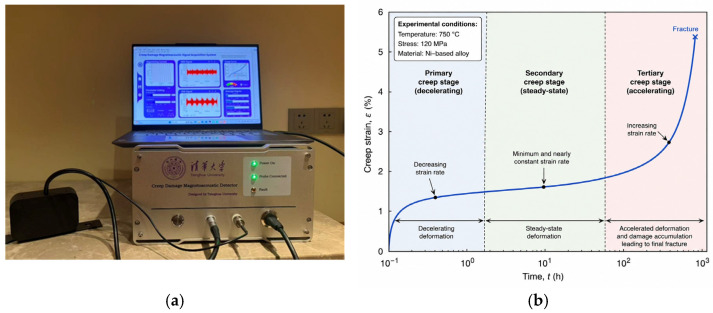
Experimental system. (**a**) Photos of independently developed experimental equipment, (**b**) representative creep curve obtained under the experimental conditions.

The experimental system adopts the MBN and MAE creep damage detection instrument developed by Tsinghua University, as shown in Figure 2a. It adopts a closed-loop magnetizing circuit and has multiple waveforms, a wide amplitude range, and a wide frequency range of magnetizing excitation output capabilities. It can stably generate an alternating magnetizing field suitable for MBN and MAE detection. The equipment can accurately output three standard magnetizing waveforms: sine wave, square wave, and triangular wave. The waveform switching is fast, and the distortion is low. Figure 2b shows the representative creep curve obtained under the experimental conditions. The curve exhibits the typical primary, secondary, and tertiary creep stages. The transition to the tertiary stage corresponds to accelerated deformation and damage accumulation before final fracture.

The magnetizing current can be continuously and accurately adjusted within the range of 0–0.6 A, with high output stability and small ripple. It has good linear driving ability in the full range, and can flexibly control the strength of the magnetization field through current intensity, covering the full range of excitation requirements from weak magnetization to strong magnetization. The excitation frequency covers an adjustable range of 5–40 Hz, with fine frequency steps and stable output. It can fully cover the optimal sensitive frequency bands of MBN and MAE signals.

The incentive module supports independent decoupling control of waveform, amplitude, and frequency parameters. After parameter setting, it can maintain a constant output for a long time, providing consistent and reliable excitation conditions for single-factor variable experiments and multi-parameter comparative experiments, ensuring the accuracy and repeatability of experimental data.

The experimental system adopts a multi-channel synchronous acquisition architecture, which can achieve parallel high-fidelity acquisition of MBN and MAE signals. The system has a maximum sampling rate of 1 MHz and can fully capture transient pulse signals and high-frequency detailed features, avoiding the loss of effective damage information. After the collected signals are processed by front-end conditioning and anti-aliasing filtering, they are transmitted in real time to the upper computer. Real-time display, online analysis, and data storage are completed through a dedicated LabVIEW 2023 program.

The system has integrated processing functions such as noise suppression, feature extraction, and parameter statistics, which can quickly output core feature parameters such as RMS value, peak value, pulse count, bandwidth, etc., providing stable and reliable data support for the analysis of the influence law of excitation parameters and the quantitative characterization of creep damage. The upper computer software can be used to set the magnetizing waveform, frequency, and magnetizing intensity, as well as to collect and process MBN and MAE signals.

## 4. Experimental Data and Analysis

### 4.1. MBN and MAE Features

MBN and MAE are both capable of characterizing the creep damage state of P92 steel, but they arise from different physical mechanisms and thus reflect different aspects of microstructural evolution during magnetization. MBN originates from the irreversible motion of magnetic domain walls overcoming pinning sites such as dislocations, precipitates, and grain boundaries, making it sensitive to microstructural changes and internal stress variations. In this study, the selected MBN features include the time-domain root mean square (RMS), peak value, pulse count, and frequency-domain bandwidth. The RMS and peak value describe the signal energy and maximum amplitude, respectively, while the pulse count and bandwidth reflect the frequency and distribution characteristics of domain wall activity.

MAE is generated by magnetostrictive deformation and the resulting elastic wave emission during magnetization. It is particularly sensitive to stress concentration, microvoids, and crack initiation and growth in P92 steel. The MAE features used here also include the RMS, peak value, pulse count, and bandwidth. These parameters characterize the overall signal intensity, discrete emission events, and frequency distribution of the acoustic response.

By extracting multidimensional MBN and MAE features, the creep damage state of P92 steel can be more comprehensively described, providing a basis for subsequent analysis of excitation parameters and damage evaluation.

### 4.2. The Influence of Waveform on MBN and MAE Signals

#### 4.2.1. Experimental Parameters and Testing Data in Different Waveform

The experiment adopts the principle of single variable control; the fixed magnetizing frequency is 5 Hz, the magnetizing current amplitude is 0.3 A, and the magnetizing waveform is only set to three types: sine wave, triangular wave (triangular wave duty cycle 50%), and square wave. MBN and MAE signals are collected for the original undamaged specimen (0% life) of P92 steel. The undamaged (0% life) specimen was mainly used to optimize the measurement parameters and establish the baseline response. The actual creep damage evaluation was performed by comparing specimens at different life fractions, which ensures that the proposed method is applicable to damage evolution analysis. The experimental data were denoised by a fourth-order Butterworth band-pass filter (10–200 kHz), and the core characteristic parameters were extracted from MBN and MAE signals, respectively: RMS value, peak value, pulse count, and bandwidth of signals were selected. The statistical results are shown in Table 2 and Table 3.

#### 4.2.2. MBN Signal Characteristic Analysis in Different Waveform

In terms of RMS value and peak value, the signal amplitude of square wave is much higher than those of sine wave and triangle wave: the RMS value of square wave (74.2 mV) is 75 times that of sine wave (0.989 mV) and 81.8 times that of triangle wave (0.907 mV); The peak value of square wave (309 mV) is 67.7 times that of sine wave (4.57 mV) and 80.5 times that of triangular wave (3.84 mV). This difference is due to the instantaneous jump characteristics of the square wave magnetic field—when the polarity of the square wave is switched, it will produce a steep rate of change in the magnetic field, which will strongly impact the domain wall, making it quickly break through the dislocation and precipitate the resistance of equal pinning points, causing a violent domain rearrangement, and then generating a high amplitude MBN signal. The sine wave magnetic field changes continuously and smoothly, and the triangular wave changes linearly and uniformly. The driving force of the two on the domain wall is milder, the signal amplitude is low, and the difference is very small (RMS difference is only 0.082 mV).

In the aspect of pulse count, the change trend is completely opposite to the amplitude: the triangular wave is the first 35 times, the sine wave is the second 32 times, and the square wave is only 22 times. This is because the MBN pulse signal originates from the discrete irreversible jump of the domain wall, and the linear magnetic field change in the triangular wave can continuously provide stable energy for the domain wall, making it break through the pinning point one by one (each break generates a pulse). The instantaneous strong impact of the square wave will lead to multiple pinning points being broken through at one time, and the domain wall will advance in a “jumping” manner, reducing the number of pulse signals. The magnetic field change rate of the sine wave is between the two, and the pulse count is also in the middle level.

In terms of bandwidth, the square wave has the widest bandwidth (65.6 kHz), 9.8 kHz wider than the sine wave (55.7 kHz) and 10.4 kHz wider than the triangular wave (55.2 kHz). This is because the square wave contains rich high-order harmonic components. When driving the domain motion, it will excite the domain vibration in a wider frequency range, making the frequency distribution of the MBN signal more dispersed. The sine wave is a single-frequency signal, and the triangular wave contains only odd harmonics. The vibration frequency range of the magnetic domain excited by the two waves is more concentrated, so the bandwidth is narrower, and the bandwidth of the triangular wave is slightly lower than that of the sine wave due to fewer harmonic components.

#### 4.2.3. MAE Signal Characteristic Analysis in Different Waveform

In terms of RMS and peak value, the square wave still has the advantage of high amplitude: the square wave RMS (0.0976 mV) is 22.7% higher than the sine wave (0.0795 V), and 33.9% higher than the triangular wave (0.0729 V); The square wave time peak (0.788 V) is 1.7 times that of the sine wave (0.465 V) and 1.4 times that of the triangular wave (0.551 V). This result comes from the magnetostrictive effect mechanism of MAE signal—the instantaneous steep magnetic field change in square wave will make the magnetostrictive strain rate in the material increase sharply, and release the elastic wave with higher strength, so the signal amplitude is larger; The magnetic field of sine wave and triangular wave changes gently, the magnetostrictive strain rate is stable, the signal amplitude is relatively low, and the time peak (0.551 V) of triangular wave is slightly higher than that of sine wave, because the linear magnetic field change in triangular wave can continuously accumulate magnetostrictive effect, making the elastic wave energy slightly superimposed.

In the aspect of pulse count, the count difference in the three waveforms is much smaller than that of the MBN signal: the triangular wave is the first with 60 times, the sine wave is the second with 58 times, and the square wave is slightly lower with 57 times, and the difference among the three is no more than 3 times. This is because the MAE pulse signal originates from the discrete propagation of an elastic wave, and the three waveforms can stably trigger the magnetostriction effect. Only a small amount of signal superposition may occur in the strong elastic wave of the square wave, resulting in a slight reduction in the count. On the whole, the sensitivity of the pulse count of the MAE signal to the excitation waveform is significantly lower than that of the MBN signal.

In terms of bandwidth, the bandwidth of the square wave (11.6 kHz) is much narrower than that of the sine wave (95 kHz) and the triangular wave (94.5 kHz). This difference is contrary to the MBN signal, which is derived from the elastic wave propagation characteristics of MAE signal—although the high-order harmonics of the square wave are abundant, the damping effect of the material on the high-frequency elastic wave is very strong, resulting in the rapid attenuation of the high-frequency components, and the final bandwidth is greatly narrowed. The magnetic field of sine wave and triangular wave changes more smoothly, and the frequency of the excited elastic wave is more concentrated in the effective response frequency band of the material, so the bandwidth is wider, and the bandwidth difference between the two is very small (only 572 Hz).

#### 4.2.4. Influence of Magnetizing Waveform on Commonness and Difference in MBN and MAE Signals

Based on the analysis of the characteristics of MBN and MAE signals, the influence of excitation waveform on the two types of signals shows the rule of “local commonality and overall differentiation”.

At the level of common influence, only the changes in amplitude parameters show consistency: the square wave can produce the highest amplitude of MBN and MAE signals, which is the common result of domain motion and magnetostriction driven by the change in instantaneous strong magnetic field of square wave as shown in Equation (10). At the same time, the triangular wave has a high pulse count in both types of signals, and its linear magnetic field change can continuously excite the discrete signal, avoiding the interference of signal superposition effect.

In terms of difference, first, there are obvious differences in amplitude sensitivity: the amplitude sensitivity of MBN signal to waveform is much higher than that of MAE signal. The amplitude difference in MBN between square wave and other waveforms can reach tens of times, while the difference in MAE signal is only tens of percent. This is because MBN is directly dependent on the micro motion of the domain wall and is more sensitive to the rate of magnetizing change. MAE depends on the macro propagation of elastic wave, which is more obviously buffered by the material characteristics [26].

Second, the change rule of the bandwidth is completely opposite: in MBN signal, the bandwidth of square wave is the widest; However, among MAE signals, the bandwidth of square wave is the narrowest. This difference is due to the different generation mechanisms of the two types of signals—bandwidth of MBN is determined by the frequency of domain vibration. The rich harmonic components of square wave make its bandwidth wider, while the bandwidth of MAE is affected by the frequency attenuation of elastic wave propagation, and the high-frequency components of square wave are rapidly weakened by material damping, resulting in a sharp narrowing of the bandwidth.

Finally, there is a significant difference in the sensitivity of pulse counting: the sensitivity of pulse counting of MAE signal to waveform is very weak, and the count difference under the three waveforms is no more than 3 times, while the difference in MBN signal can reach 13 times, which reflects that the stability of elastic wave propagation is much higher than the micro motion process of domain wall.

To sum up, the influence of magnetizing waveform on MBN and MAE signals is significantly different. In practical application, the waveform should be selected according to the detection requirements of signal types: if it is necessary to obtain high amplitude or wide band signals of MBN, the square wave is preferred. If MAE high amplitude signal is required and narrow bandwidth is acceptable, square wave is also applicable. If high pulse counting of two kinds of signals is required, triangular wave is the best choice.

Additionally, the high MAE amplitude in the square wave and the high pulse count in the triangle wave in this experiment are fundamentally driven by differences in the magnetostriction strain rate from Equations (11)–(13). The MAE signal originates from the instantaneous micro-strain release caused by magnetic domain motion, while the pulse count corresponds to the discrete number of micro-strain events. This result validates the “strain rate-discreteness” mechanism of magnetostriction, where the MAE signal characteristics essentially represent the combined manifestation of temporal distribution and spatial discretization of magnetic domain motion.

### 4.3. The Influence of Frequency on the MBN and MAE Signals

#### 4.3.1. Experimental Parameters and Testing Data in Different Frequency

**Figure 3:**
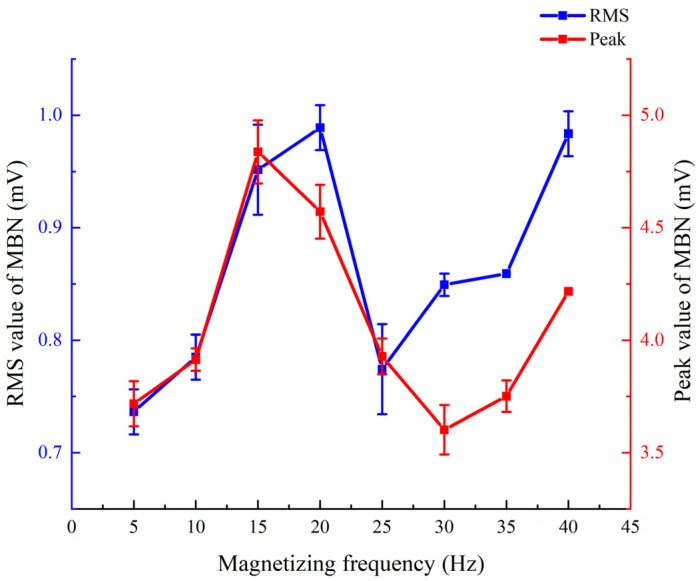
MBN signals time domain characteristics under different magnetizing frequencies.

**Figure 4:**
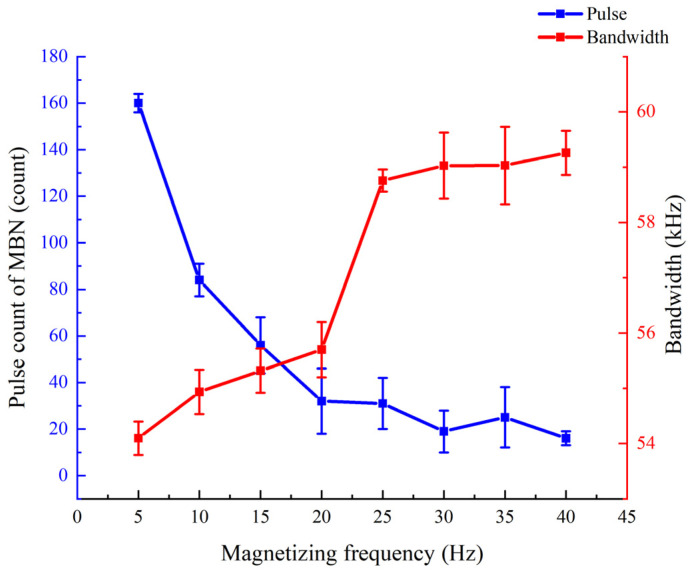
MBN signals frequency domain characteristics under different magnetizing frequencies.

**Figure 5:**
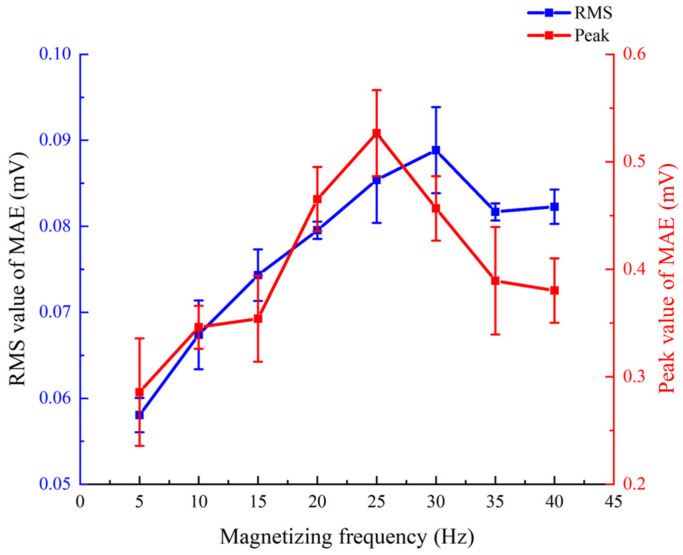
MAE signals time domain characteristics under different magnetizing frequencies.

**Figure 6:**
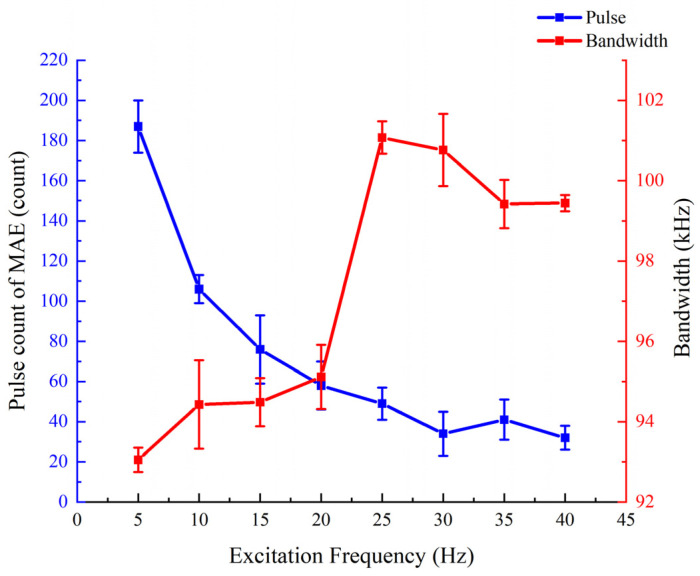
MAE signals frequency domain characteristics under different magnetizing frequencies.

This experiment also adopts the principle of single variable control. The fixed magnetizing waveform is sine wave, and the amplitude is 0.3 A. Only the frequency (5 Hz, 10 Hz, 15 Hz, 20 Hz, 25 Hz, 30 Hz, 35 Hz, 40 Hz) is adjusted. MBN and MAE signals are collected from the original undamaged sample of P92 steel. After the experimental data is denoised by the 4th order Butterworth bandpass filter (10–200 kHz), the RMS value, peak value, pulse count and bandwidth of MBN and MAE signals are extracted as the core characteristic parameters respectively. All data are the average values of three parallel experiments. The statistical results are shown in Figure 3, Figure 4, Figure 5 and Figure 6.

#### 4.3.2. MBN Signal Characteristic Analysis in Different Frequency

In terms of RMS and peak value, they show a similar fluctuation trend: with the magnetizing frequency rising from 5 Hz to 20 Hz, time RMS gradually rises from 0.736 mV to 0.989 mV (peak), and peak value rises from 3.72 mV to 4.84 mV (at 15 Hz) and then decreases slightly. After 20 Hz, RMS first decreased to 0.77 mV (25 Hz), and then fluctuated back to 0.98 mV (40 Hz), while peak value showed a downward trend. This change is due to the response characteristics of the domain wall—as low as 20 Hz, the increase in frequency increases the rate of change in the magnetizing field, which is easier to drive the domain wall to break through the pinning point and increase the signal amplitude. After 20 Hz, the frequency exceeded the response frequency of the domain wall, and some domain walls could not keep up with the rhythm of the magnetizing field, resulting in the amplitude fluctuation falling back. Only at 40 Hz, due to the superposition of high-frequency harmonics, the amplitude rose slightly.

In the aspect of pulse count, the change trend is significantly negatively correlated with the amplitude: the pulse count reaches 160 times (maximum) at 5 Hz, and continues to decline as the frequency rises to 40 Hz, reaching only 16 times at 40 Hz. This is because the MBN pulse originates from the discrete jump of the domain wall, the magnetizing field change period is long at low frequency, and the domain wall has enough time to break through the pinning points one by one, producing a large number of pulses. With the increase in frequency, the variation period of the magnetizing field is shortened, the domain walls have no time to respond one by one, and move in the way of “batch breakthrough”, and the pulse count is greatly reduced.

In terms of frequency spectral centroid (the center of gravity of the spectrum, which reflects the signal frequency concentration trend), the overall trend continues to rise: from 54 kHz at 5 Hz to 59 kHz at 40 Hz, with only a small fluctuation before 20 Hz. This is because the increase in magnetizing frequency will introduce higher-order harmonics, which will shift the frequency distribution of the MBN signal to the high-frequency band, and the spectrum center of gravity will rise. Moreover, the harmonic components will be richer at high frequencies, and the rising trend of the spectrum center of gravity will be more stable.

#### 4.3.3. MAE Signal Characteristic Analysis in Different Frequency

In terms of RMS value and peak value, they show a core trend of “first rising and then falling”: with the excitation frequency rising from 5 Hz to 25 Hz, RMS value gradually rises from 0.058 to 0.085 (peak), and peak value rises from 0.29 mV to 0.53 mV (maximum at 25 Hz). After 25 Hz, RMS value fluctuated slightly, and peak value decreased from 0.53 mV to about 0.38 mV. This rule is derived from the magnetostrictive effect mechanism of MAE—as low as 25 Hz, the increase in frequency increases the rate of change in the magnetizing field, the response strength of magnetostrictive strain in the material increases, and the signal amplitude continues to rise. After 25 Hz, the frequency exceeds the response frequency of material magnetostriction, and the growth rate of strain rate slows down. At the same time, the damping attenuation of elastic wave at high frequency intensifies, resulting in the decline of signal amplitude.

In the aspect of pulse count, the change trend is significantly negatively correlated with the amplitude: the pulse count reaches 187 times (maximum) at 5 Hz, and continues to decline as the frequency rises to 40 Hz, only 32 times at 40 Hz. This is because MAE pulse originates from the discrete propagation of magnetostrictive elastic wave, the magnetizing field has a long period of change at low frequency, and the elastic wave can be released stably in batches, producing a large number of pulses. When the frequency increases, the variation period of the magnetizing field is shortened, the elastic wave is prone to superposition or continuous propagation, and the pulse count is reduced.

In terms of bandwidth, the overall trend is “fluctuating after rising”: it gradually rises from 93 kHz at 5 Hz to 101 kHz at 25 Hz (peak), and then falls back to about 99 kHz after 25 Hz. This change is related to the frequency distribution of MAE signal—as low as 25 Hz, the increase in frequency introduces more high-order harmonics, the frequency distribution range of elastic wave widens, and the bandwidth increases. After 25 Hz, the high-frequency elastic wave decays rapidly due to damping, the signal frequency distribution is concentrated again, and the bandwidth decreases slightly and remains stable.

#### 4.3.4. Effect of Magnetizing Frequency on MBN and MAE Signals

Based on the analysis of the characteristics of MBN and MAE signals under sine wave and 0.3 A magnetization, the influence of magnetizing frequency on the two types of signals presents the core conclusion of “trend correlation and mechanism differentiation.”

From the change trend of parameters, there is a clear correlation between the two types of signals: first, the amplitude parameters (RMS, Peak) show the law of “first rising and then falling” with the increase in frequency, and only the response frequency is slightly different (MBN is about 20 Hz, MAE is about 25 Hz). The second is that the pulse count continuously decreases with the increase in frequency, and the pulses are more dense at low frequency and sparse at high frequency. Third, the frequency related parameters (spectrum barycenter, bandwidth) are “stable after rising” with the increase in frequency, and high frequency broadens the frequency distribution range of the signal.

From the perspective of mechanism differentiation, the difference between the two types of signals stems from the different generation principles: MBN signals are more sensitive to frequency (the amplitude fluctuation is more obvious after 20 Hz) and the rising trend of the spectrum center of gravity is more sustained, which depends on the micro motion of the domain wall. The MAE signal depends on the macro propagation of elastic wave, and is more significantly affected by the damping characteristics of the material (the amplitude drop is more obvious after 25 Hz), and the fluctuation amplitude of bandwidth is larger.

Combined with the actual testing requirements, under the sine wave and 0.3 A magnetizing conditions: if it is necessary to obtain the high amplitude signals of MBN and MAE, it is recommended to select the magnetizing frequency of 20–25 Hz. If high pulse counting is required to improve the signal discreteness, it is recommended to select a low frequency of 5–10 Hz. If the signal with wide frequency distribution is required to cover more characteristic frequency bands, it is recommended to select the frequency range of 20–25 Hz.

### 4.4. The Influence of Magnetizing Amplitude on the MBN and MAE Signals

#### 4.4.1. Experimental Parameters and Testing Data in Different Magnetizing Amplitude

**Figure 7:**
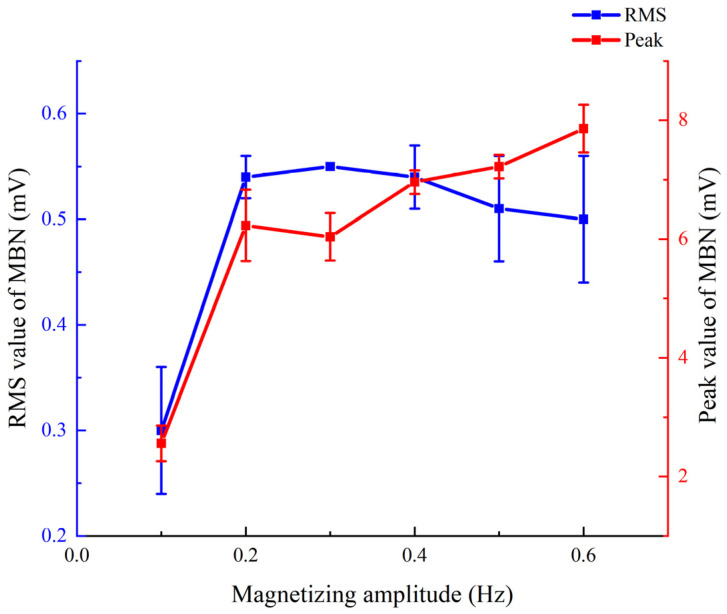
MBN signals time domain characteristics under different magnetizing amplitudes.

**Figure 8:**
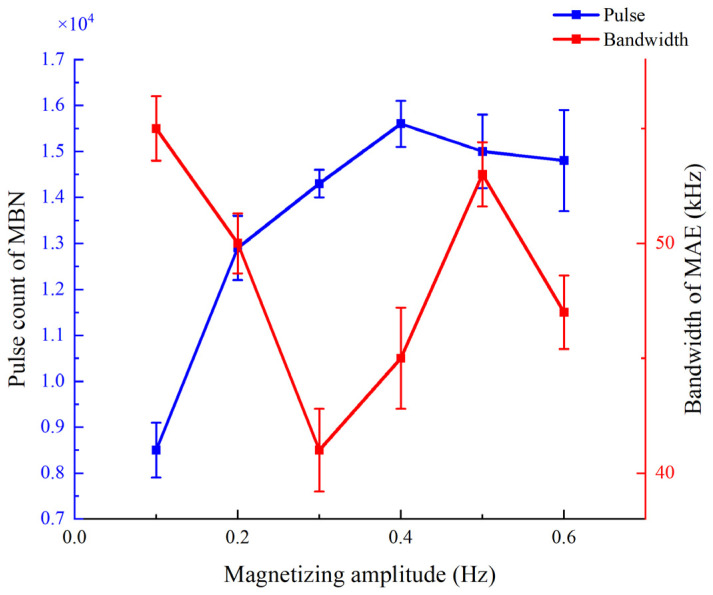
MBN signals frequency domain characteristics under different magnetizing amplitudes.

**Figure 9:**
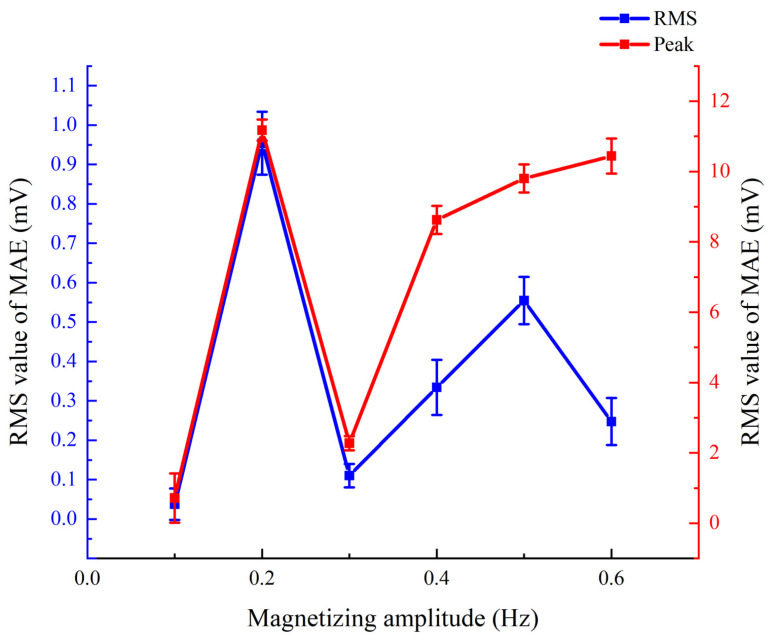
MAE signals time domain characteristics under different magnetizing amplitudes.

**Figure 10:**
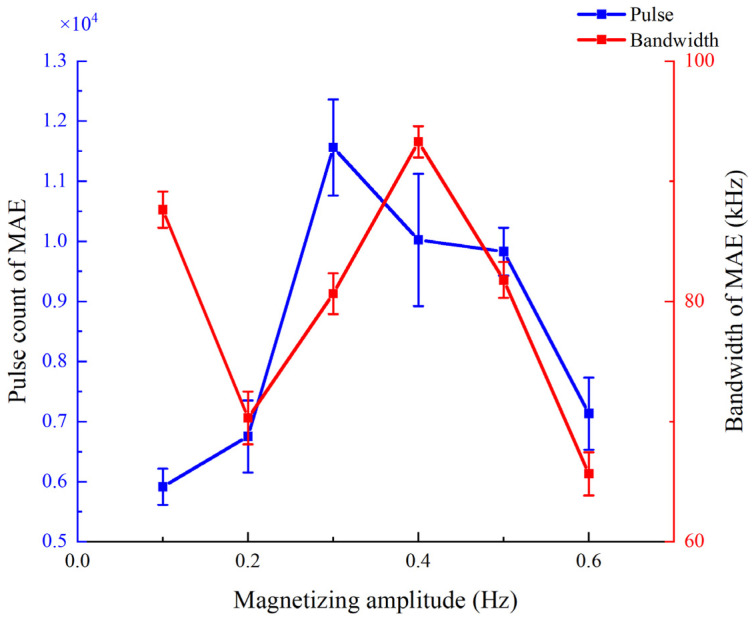
MAE signals frequency domain characteristics under different magnetizing amplitudes.

The magnetizing waveform is a sine wave, and the frequency is 20 Hz. Only the magnetizing amplitude (0.1 A, 0.2 A, 0.3 A, 0.4 A, 0.5 A, 0.6 A) is adjusted. MBN and MAE signals are collected from the original undamaged samples of P92 steel. The experimental data were denoised by a fourth-order Butterworth bandpass filter (10–200 kHz), and the RMS value, peak value, pulse count, and bandwidth of MBN and MAE signals are taken as the core characteristic parameters. All data are the average values of three parallel experiments. The results are shown in Figure 7, Figure 8, Figure 9 and Figure 10.

#### 4.4.2. MBN Signal Characteristic Analysis in Different Magnetizing Amplitude

Based on the MBN signal characteristics in Figure 7 and Figure 8, the influence of magnetizing amplitude on MBN parameters presents a phased rule of “first rising and then stabilizing”.

In terms of RMS value and peak value, within the range of 0.1 A to 0.4 A, the RMS value gradually increased from 0.30 mV to 0.54 mV (peak), and the peak value increased from 2.45 mV to 7.19 mV. After 0.4 A, the RMS value fell slightly and remained in the range of 0.52–0.54 mV, while the peak value continued to rise slowly. This change is due to the driving force mechanism of the domain wall—at low amplitude, the magnetizing field strength is insufficient, the domain wall can only break through a few pinning points, and the signal amplitude is low. With the amplitude increasing to 0.4 A, the magnetizing field driving force is enough to stimulate most of the domain wall motion, and the amplitude is close to saturation; After 0.4 A, the magnetizing field strength continues to increase, but the domain wall motion has reached its limit, and only the peak value increases slightly due to the violent vibration of local domains.

In terms of pulse count, within the range of 0.1 A to 0.4 A, the count continued to rise from 8.53 kHz to 15.6 kHz (peak). After 0.4 A, the count dropped slightly and stabilized in the range of 15.2–15.5 kHz. This is because the increase in excitation amplitude will enhance the driving force of the magnetizing field on the domain wall, so that more pinning points are broken through, and the number of pulse signals increases. When the amplitude reaches 0.4 A, the number of domain walls that can be excited approaches the upper limit, and the pulse count enters a stable stage.

In terms of bandwidth, the overall trend is “down first and then fluctuating”: within the range of 0.1 A to 0.3 A, the bandwidth decreases from 53.17 kHz to 41.44 kHz (valley value). After 0.3 A, the bandwidth fluctuation rebounded and remained in the range of 45–52 kHz. This is because the frequency distribution of domain wall motion is more dispersed and the bandwidth is wider at low amplitude. With the increase in amplitude, the motion of domain wall is gradually synchronized, the frequency distribution is more concentrated, and the bandwidth is narrowed. After 0.3 A, the frequency distribution is dispersed again and the bandwidth fluctuation rises again due to the asynchronous vibration of some domain walls.

#### 4.4.3. Characteristic Analysis of the MAE Signal in Different Magnetizing Amplitude

Based on the MAE signal characteristics in Figure 9 and Figure 10, the influence of magnetizing amplitude on MAE parameters is characterized by “fluctuating variation”, which is significantly different from the MBN signal Law.

In terms of RMS value and peak value, both showed a non-monotonic fluctuation trend: RMS value had a local peak at 0.2 A (0.954 mV) and 0.5 A (0.555 mV), while peak value continued to rise with the increase in amplitude, reaching 10.442 mV at 0.6 A. This difference is due to the magnetostrictive effect of MAE—the magnetostrictive strain has a non-linear relationship with the magnetic field strength, and the uneven distribution of stress in the material makes the signal amplitude fluctuate. The peak value increases continuously with the increase in magnetizing field strength, which reflects the continuous improvement of elastic wave release intensity in the local stress concentration region. In addition, the amplitude response of MAE is also closely related to the distribution of the internal stress field and microscopic heterogeneity of the material: in local stress concentration regions, the magnetostrictive strain rate may increase rapidly, leading to a sharp rise in the local elastic-wave excitation intensity and thus an increase in the peak value; whereas, in regions with relatively uniform stress distribution or stronger constraints, the release efficiency of elastic waves is lower, so the peak growth is limited. At the same time, material damping and the initiation/early propagation of microcracks can suppress or disperse the energy output of high-frequency components, causing the peak value to exhibit a non-monotonic relationship with amplitude.

In terms of pulse count, the count showed the fluctuation characteristics of “sudden drop—sudden rise”: the count reached 5915 at 0.1 A, dropped to 339 at 0.2 A, rebounded to 11,561 at 0.3 A, dropped to 221 again at 0.4 A, and rebounded to 8131 at 0.6 A. This is because the MAE pulse is related to the propagation path of the elastic wave. At low amplitude, elastic wave propagation is more dispersed, and the pulse count is higher. When the amplitude is medium, the elastic wave is easy to stack, and the count drops sharply. At high amplitude, the local stress concentration area increases, the elastic wave disperses and propagates, and the count rises again. At low amplitudes, the scattering effect of microcracks and local defects on elastic waves dominates, resulting in a more dispersed energy release and a lower likelihood of high pulse counts; in the medium-amplitude range, the superposition effect of elastic waves induced by local stress concentration may lead to “peak concentration,” causing a sudden drop in pulse count. In the high-amplitude range, as the stress field redistributes and the energy transmission is reorganized, the propagation paths of elastic waves increase and become more constrained, while some regions transfer energy into other modes, leading to a rebound in pulse count. In addition, scattering at interfaces and grain boundaries, acoustic mode coupling, and microstructural evolution within the material (such as phase transformation and dislocation redistribution) can also have a significant impact on pulse count.

In terms of bandwidth, the overall trend is “fluctuating downward”: within the range of 0.1 A to 0.4 A, the bandwidth rises from 87.63 kHz to 93.29 kHz (peak). After 0.4 A, the bandwidth continues to fall back to 65.64 kHz. This is because the frequency distribution of the elastic wave is wide at low amplitude. With the increase in amplitude, the elastic wave propagation gradually synchronizes, and the bandwidth first increases and then decreases. At high amplitude, the damping effect of the material is enhanced, the attenuation of high-frequency elastic wave is intensified, and the bandwidth is narrowed. Local stress-field heterogeneity and acoustic scattering from grains and grain boundaries alter the relative intensity of propagating modes, causing certain high-frequency components to be strongly dissipated during propagation and thus narrowing the bandwidth; meanwhile, if the local high-frequency vibration induced by magnetostrictive coupling is impedance-mismatched with the material, energy redistribution will occur, thereby affecting the bandwidth.

#### 4.4.4. Conclusion of the Influence of Magnetizing Amplitude on MBN and MAE Signals

Based on the analysis of the characteristics of the two types of signals, the influence of magnetizing amplitude on MBN and MAE signals shows significant differences.

The response of the MBN signal to magnetizing amplitude is more stable, and the variation in parameters is mainly “rise first and then stabilize”. The optimal amplitude of MBN excitation current is 0.4 A (both amplitude and pulse count are saturated, and the bandwidth is stable). However, the response of MAE signal to magnetizing amplitude is “fluctuating”, and there is no uniform optimal amplitude range for parameters, which should be selected in combination with specific testing requirements (0.1 A, 0.3 A or 0.6 A for high pulse count; 0.6 A for high peak value).

This difference is due to the generation mechanism of two kinds of signals: MBN depends on the micro motion of the domain wall, and the response law is more uniform; MAE depends on the macro propagation of the elastic wave and is more significantly affected by the material stress distribution, so the parameter changes are more complex. Based on the above observations, the proposed framework helps explain the qualitative evolution of the MBN and MAE responses. However, the present study is not intended to develop a comprehensive predictive model. Instead, it emphasizes experimental evidence and statistical comparison, which together constitute the main contribution of the work.

### 4.5. Quantitative Validation of the Theoretical Model Against Experimental MBN/MAE Data

To quantitatively assess the proposed theoretical model, its predictions were compared with the experimentally measured MBN and MAE responses of P92 steel under different magnetization waveforms, amplitudes, and frequencies. The comparison focused on the main signal descriptors, including RMS, peak value, pulse count, and bandwidth.

As shown in Table 4, the model reproduces the principal experimental trends with good consistency. In particular, sine-wave excitation yields the most stable response, whereas square-wave excitation produces higher signal intensity but larger fluctuations. The predicted effects of magnetization amplitude and frequency also agree well with the measurements, confirming that the model captures the dominant magneto-mechanical coupling behavior within the tested range. Minor deviations are observed in MAE bandwidth, which may be attributed to wave attenuation, material damping, and local microstructural heterogeneity during acoustic emission propagation. Nevertheless, the relative errors remain acceptable, indicating that the model has sufficient predictive capability for magnetization parameter optimization.

Overall, the quantitative comparison demonstrates that the proposed model provides a reliable description of the MBN/MAE responses of P92 steel under creep damage conditions and offers a solid basis for interpreting the experimental results.

## 5. Conclusions

This study focuses on the optimization of magnetizing parameters for creep damage detection of P92 steel. By analyzing the characteristics of MBN and MAE signals under different waveforms, frequencies, and amplitudes, the magnetizing parameter combination that gives the best signal response under the tested conditions is finally determined. The specific conclusions are as follows:

In the selection of magnetizing waveform, the sine wave shows the most stable signal response. Compared with square wave and triangle wave, sine wave has a more stable pulse count and a more concentrated bandwidth distribution for the MBN signal, and the proportion of effective signal is significantly higher; for the MAE signal, its amplitude and pulse count fluctuate less, and the overall signal stability is better than the high-noise characteristics of square wave and the low-amplitude characteristics of triangular wave, which allows both types of signals to be detected with comparable reliability.

In the selection of magnetizing amplitude, 0.3 A gives the best signal response under the condition of a sine wave and 20 Hz. For MBN signals, the amplitude of 0.3 A is close to saturation, the pulse count is sufficient, and the bandwidth remains in a narrow range, which not only avoids the problem of insufficient signal strength at low amplitude but also avoids signal saturation at high amplitude; for the MAE signal, the pulse count corresponding to 0.3 A reaches 11,561 times.

In the selection of magnetizing frequency, 20 Hz matches the response characteristics of the two types of signals. Under the condition of sine wave and 0.3 A, 20 Hz gives the best balance between amplitude and pulse count for the MBN signal, the distribution of the spectrum gravity center is concentrated, and the signal characteristics are the most identifiable; for the MAE signal, the amplitude, pulse count and bandwidth at 20 Hz are within the selected range, which can effectively excite the magnetostrictive effect, reduce damping attenuation during elastic wave propagation, and ensure effective signal transmission.

Based on the above analysis, a sine wave, 0.3 A, and 20 Hz are selected as the magnetizing parameter combination for P92 steel creep damage detection under the tested conditions. Under this combination, the amplitude of MBN and MAE signals is intermediate, the pulse count is sufficient, and the bandwidth distribution is stable, which ensures clear identification of signal characteristics and takes into account the stability and repeatability of the detection process, providing a reliable signal basis for the quantitative assessment of creep damage of P92 steel.

Note on limitations: The conclusions regarding microstructural evolution inferred from MBN/MAE signals in this study are based solely on fracture-surface observations and signal measurements of creep-damaged specimens. No metallographic verification was performed in this work. Metallographic analyses (e.g., SEM/EBSD/TEM) on creep-tested samples are planned for future work to directly correlate microstructural changes with MBN/MAE responses and to refine the mechanistic interpretation. In practical applications, the magnetizing waveform should be selected according to the detection purpose. Square-wave magnetization is more suitable for obtaining higher MBN amplitude and wider bandwidth, while triangular-wave magnetization is better for achieving higher pulse counts. For MAE signals, square-wave magnetization can provide higher amplitude, but the sensitivity to waveform is weaker than that of MBN.

## Figures and Tables

**Table 1:** Main element composition and mass fraction of P92 steel.

Component	Percentage
Fe	balance
Cr	8.5–9.5%
Mo	0.3–0.6%
W	1.5–2.0%
V	0.15–0.25%
Nb	0.04–0.09%
C	0.07–0.13%
N	0.03–0.07%

**Table 2:** Time and frequency domain characteristics of the MBN signal in ten tests.

Magnetizing Waveform	RMS Value (mV)	Peak Value (mV)	Pulse Count	Bandwidth (kHz)
Sine wave	0.989 ± 0.031	4.57 ± 0.18	32.0 ± 1.7	55.7 ± 1.2
Triangular wave	0.907 ± 0.027	3.84 ± 0.15	35.0 ± 1.9	55.2 ± 1.3
Square wave	74.2 ± 2.6	309 ± 9.8	22.0 ± 1.1	65.6 ± 1.5

**Table 3:** Time and frequency domain characteristics of the MAE signal in ten tests.

Magnetizing Waveform	RMS Value (V)	Peak Value (V)	Pulse Count	Bandwidth (kHz)
Sine wave	0.0795 ± 0.0026	0.465 ± 0.015	58.0 ± 2.1	95.0 ± 3.0
Triangular wave	0.0729 ± 0.0023	0.551 ± 0.017	60.0 ± 2.0	94.0 ± 2.8
Square wave	0.0976 ± 0.0031	0.788 ± 0.025	57.0 ± 1.8	12.0 ± 0.7

**Table 4:** Quantitative comparison between experimental and predicted MBN/MAE features.

Signal	Experimental Value	Predicted Value	Relative Error (%)
Sine, MBN RMS (mV)	0.989	0.972	1.72
Sine, MBN Peak (mV)	4.57	4.49	1.75
Sine, MBN Pulse Count	32	31	3.13
Sine, MBN Bandwidth (kHz)	55.7	54.9	1.44
Sine, MAE RMS (V)	0.0795	0.0782	1.64
Sine, MAE Peak (V)	0.465	0.458	1.51
Sine, MAE Pulse Count	58	57	1.72
Sine, MAE Bandwidth (kHz)	95.0	93.6	1.47
Sine, MBN RMS (mV)	74.2	71.8	3.23
Sine, MAE RMS (V)	0.0976	0.0948	2.87

## Data Availability

The data presented in this study are available on request from the corresponding author. The project is funded by National Natural Science Foundation of China. Due to data privacy, data is only been provided only if it’s on request.
